# Response to Commentary on “Pedal Medial Arterial Calcification in Diabetic Foot Ulcers: A Significant Risk Factor of Amputation and Mortality”

**DOI:** 10.1111/1753-0407.70070

**Published:** 2025-03-04

**Authors:** Lihong Chen, Dawei Chen, Hongping Gong, Chun Wang, Yun Gao, Yan Li, Weiwei Tang, Panpan Zha, Xingwu Ran

**Affiliations:** ^1^ Department of Endocrinology & Metabolism, West China Hospital Sichuan University Chengdu China; ^2^ Innovation Center for Diabetic Foot, Diabetic Foot Care Center, West China Hospital Sichuan University Chengdu China; ^3^ International Medical Center Ward, Department of General Practice, West China Hospital Sichuan University Chengdu China; ^4^ Department of Clinical Research Management, West China Hospital Sichuan University Chengdu China

Dear Editor,

We appreciate the authors for their insightful commentary and for recognizing our manuscript [1] as a valuable contribution that provides crucial insights into the relationship between pedal MAC, the risk of amputation, and mortality in patients with diabetic foot ulcers (DFUs).

We concur that a more sophisticated classification system, such as SINBAD or WIfI systems, could enhance the comprehension of the severity and risk associated with DFUs and improve communication among healthcare professionals. However, considering its simplicity and practicality, the Wagner wound classification system remains internationally recognized and widely utilized.

Regarding laboratory markers, inflammatory biomarkers including erythrocyte sedimentation rate (ESR), C‐reactive protein (CRP), Neutrophil‐to‐lymphocyte ratio (NLR), platelet‐to‐lymphocyte ratio (PLR), and systemic inflammatory response index (SIRI) exhibit significant variability. Specifically, in patients with diabetic foot infections, these inflammatory markers are markedly elevated. Consequently, they serve as indicators of acute infection but are not suitable for assessing long‐term prognosis, such as amputation risk or mortality. Additionally, laboratory markers such as magnesium, zinc, and vitamin B12 are not routinely evaluated in clinical practice. Further research could explore the potential associations between these markers and the prognosis of DFUs.

Because of the longitudinal nature of the study design, participants were followed up over an extended period. The hypoglycemic medications prescribed may have varied over time, making it challenging to incorporate both the medications and their dosages into the analysis. Regarding infections and antibiotics, diabetic foot infection is indeed a recognized risk factor for amputation and short‐term mortality. However, based on our previous meta‐analysis, the primary long‐term causes of mortality include cardiovascular diseases, infections (such as sepsis, respiratory infections, and foot infections), and cancers [2].

We acknowledge that patients with DFUs may concurrently suffer from autoimmune disorders, psychiatric conditions, and malignancies. These comorbidities elevate the risk of amputation and adverse outcomes [3]. However, in this study, we excluded individuals with these comorbidities prior to analysis.

With respect to socioeconomic status and educational attainment, it is well‐established that a lower socioeconomic status constitutes a substantial risk factor for amputation among patients with diabetes and peripheral artery disease [4]. Socioeconomic status, social capital, and medical challenges significantly impede the effective management and prevention of DFUs [5]. Enhanced government intervention is imperative to ensure equitable access to health resources. Additionally, a history of ulceration and prior amputations is a critical risk factor for future amputations and mortality. We concur that these elements must be considered in comprehensive patient care strategies.

Lastly, we fully concur that the follow‐up method of telephone interviews inherently possesses certain limitations. To address this, our diabetic foot care center plans to establish a diabetic foot registry cohort in the future, which will yield more precise and reliable follow‐up data.

In conclusion, we would like to express our gratitude to the authors for their insightful and constructive critiques of our manuscript. In future research endeavors, we aim to incorporate several laboratory markers and risk factors in order to enhance both the management and future investigation of DFUs.

## Conflicts of Interest

The authors declare no conflicts of interest.
